# Availability and Use of Molecular Microbiological and Immunological Tests for the Diagnosis of Tuberculosis in Europe

**DOI:** 10.1371/journal.pone.0099129

**Published:** 2014-06-12

**Authors:** Marc Tebruegge, Nicole Ritz, Karsten Koetz, Antoni Noguera-Julian, James A. Seddon, Steven B. Welch, Maria Tsolia, Beate Kampmann

**Affiliations:** 1 Academic Unit of Clinical & Experimental Sciences, Faculty of Medicine, University of Southampton, Southampton, United Kingdom; 2 Department of Paediatric Infectious Diseases & Immunology, University Hospital Southampton NHS Foundation Trust, Southampton, United Kingdom; 3 Institute for Life Sciences, University of Southampton, Southampton, United Kingdom; 4 Department of Paediatrics, The University of Melbourne, Parkville, Australia; 5 Infectious Diseases Unit, University Children's Hospital Basel, Basel, Switzerland; 6 Department of Paediatrics, Sahlgrenska University Hospital, Gothenburg, Sweden; 7 Department of Paediatrics, Hospital Sant Joan de Déu - Universitat de Barcelona, Barcelona, Spain; 8 Academic Department of Paediatrics, Faculty of Medicine, Imperial College, London, United Kingdom; 9 Birmingham Chest Clinic, Heart of England NHS Foundation Trust, Birmingham, United Kingdom; 10 Second University Department of Pediatrics, National and Kapodistrian University of Athens School of Medicine, ''P. & A. Kyriakou'' Children's Hospital, Athens, Greece; 11 Medical Research Council (MRC) Unit The Gambia, Banjul, The Gambia; McGill University, Canada

## Abstract

**Introduction:**

Currently only limited data exist regarding the availability and clinical use of molecular and immunological tests for tuberculosis (TB) in the European setting.

**Methods:**

Web-based survey of Paediatric-Tuberculosis-Network-European-Trialsgroup (ptbnet) and Tuberculosis-Network-European-Trialsgroup (TBnet) members conducted June to December 2013. Both networks comprise clinicians, microbiologists, epidemiologists and researchers predominately based in Europe.

**Results:**

191 healthcare professionals from 31 European countries participated. Overall, 26.8% of respondents did not have access to the Xpert MTB/RIF assay; only 44.6% had access to the assay in-house. However, a substantial proportion had access to other commercial and/or non-commercial PCR-based assays for TB (68.8% and 31.8%, respectively). Only 6.4% did not have access to any PCR-based assays for TB. A large proportion of participants with access to the Xpert MTB/RIF assay had used it for the analysis of non-respiratory samples [pleural fluid: 36.5%, gastric aspirates: 34.7%, cerebrospinal fluid: 34.7%, stool samples: 4.3%, blood/serum: 2.6%, ‘other samples’ (which included biopsy/tissue samples, lymph node aspirates, joint aspirates and urine samples): 16.5%]. Regarding interferon-gamma release assays, a greater proportion of respondents had access to the QuantiFERON-TB Gold assay (84.7%) than to the T-SPOT.*TB* assay (52.2%).

**Conclusions:**

Both immunological and molecular TB tests are widely available across Europe. The QuantiFERON-TB Gold assay is more widely used than the T-SPOT.*TB* assay, which may reflect the difficulties of integrating an ELISPOT assay into the routine laboratory setting. Although Xpert MTB/RIF assays are optimised and solely licensed for the analysis of sputum samples, in clinical practice they are commonly used for non-respiratory samples. Further research is needed to establish how current molecular TB tests impact on patient care and outcome in the routine clinical setting.

## Introduction

Detection of *Mycobacterium tuberculosis* by microbiological methods remains the gold standard for the diagnosis of tuberculosis (TB) disease in humans, also referred to as active TB. However, traditional solid and liquid culture methods can take several weeks to produce a positive result [1], [2]. In recent years, several commercial molecular assays for the detection of *M. tuberculosis*, based on the polymerase chain reaction (PCR) principle, have become available, which have the advantage of potentially significantly shortening the time needed to confirm suspected TB disease [1], [3]–[9].

In December 2010 the World Health Organization (WHO) issued their official endorsement of the Xpert MTB/RIF assay (Cepheid; Sunnyvale, CA, U.S.) for the diagnosis of TB [10]. The assay is based on a qualitative, nested real-time PCR, which allows the detection of *M. tuberculosis* complex in clinical samples, and simultaneously detects mutations in the *rpoB* gene, which are associated with rifampicin resistance [1], [4], [11], [12].

To facilitate uptake and implementation of the Xpert MTB/RIF assay globally, concessional pricing was negotiated for more than 140 low- and middle-income countries. According to the WHO, as of September 2013 a total of 1,843 GeneXpert instruments and more than 4.2 million Xpert MTB/RIF cartridges have been procured by 95 countries eligible for concessional pricing [13]. However, while the WHO collects and regularly publishes data on the progress of the global Xpert MTB/RIF roll-out, this exclusively comprises data from countries eligible for concessional pricing, which excludes all Western, and the majority of Northern and Southern European countries. Therefore, only very limited data regarding the availability and clinical use of Xpert MTB/RIF assays and other molecular assays in the European setting are currently available.

Interferon-gamma release assays (IGRA) were licensed for clinical use in 2001 [14]. IGRA are solely licensed for the diagnosis of latent TB infection, and rely on the detection of interferon-gamma produced by sensitised T cells in response to stimulation with *M. tuberculosis*-specific antigens [15]–[20]. Currently two IGRA are commercially available, the QuantiFERON-TB Gold assay (Cellestis/Qiagen; Carnegie, Australia) and the T-SPOT.*TB* assay (Oxford Immunotech; Abingdon, United Kingdom), which are based on ELISA and ELISPOT formats, respectively [17]. To date, there are few data on the availability of IGRA across Europe, since the WHO does not routinely collect data related to these immunoassays.

The aims of this study were to determine the availability of molecular microbiological and immunological diagnostic tests for TB in European countries, to establish how these tests are being used in clinical practice, and to determine how molecular tests are currently being funded across Europe.

## Methods

### Participants

A web-based survey was conducted among the members of the Paediatric Tuberculosis Network European Trialsgroup (ptbnet) and the Tuberculosis Network European Trialsgroup (TBnet) over a 6-month-period (June to December 2013). Both networks comprise clinicians, microbiologists, epidemiologists and researchers, with the majority (86%; n = 545) of the members being based in Europe (for further details see: http://www.tb-net.org/index.php/about-us; http://www.tb-net.org/index.php/about-us/tbnet-members; http://www.tb-net.org/index.php/ptbnet) [21]. Network members were contacted by email and invited to complete the survey online.

### Definitions

For the purpose of this study ‘Europe’ was defined according to the United Nations Statistics Division definition, which currently includes 42 countries (for further details see: http://unstats.un.org/unsd/methods/m49/m49regin.htm#europe). ‘Eastern Europe’ was defined as a geographical region comprising the following countries: Albania, Belarus, Bosnia and Herzegovina, Bulgaria, Croatia, the Czech Republic, Estonia, Hungary, Latvia, Lithuania, Macedonia, Moldova, Montenegro, Poland, Romania, the Russian Federation, Serbia, Slovakia, Slovenia and Ukraine.

### Survey instrument

The online survey instrument was developed and initially trialled individually by all authors to identify potential technical issues. Following this, the survey instrument was trialled by five ptbnet members based in different European countries who were not part of the study team. No technical issues were identified at this stage, but minor adjustments were made to the question wording based on the feedback provided. Table S1 (supplementary digital contents) provides a summary of the final survey instrument.

### Statistical analysis and data deposition

STATA (Version 12; StataCorp; College Station, TX, US) and Prism (Version 5.0; GraphPad; La Jolla, CA, US) were used for data analyses and construction of the figures. Fisher's exact tests were used to assess differences between subgroups of participants. P-values<0.05 were considered to be statistically significant. The original data are accessible via the University of Southampton ePrints digital repository (at http://eprints.soton.ac.uk/364424/; DOI: 10.5258/SOTON/364424).

### Ethics approval

According to current UK National Research Ethics Service (NRES) regulations, Research Ethics Committee review is not required for research involving healthcare staff recruited as research participants by virtue of their professional role (Governance Arrangements for Research Ethics Committees, paragraph 2.3.13). Participation in the survey was voluntary. No identifying/personal information was collected. Participants were aware that they were participating in research, and that the results would be published.

## Results

A total of 191 healthcare professionals from 31 European countries (Austria, Belarus, Belgium, Bulgaria, Croatia, the Czech Republic, Denmark, Finland, France, Germany, Greece, Hungary, Italy, Lithuania, Macedonia, Moldova, the Netherlands, Norway, Poland, Portugal, Romania, the Russian Federation, Serbia, Slovakia, Slovenia, Spain, Sweden, Switzerland, Turkey, Ukraine, United Kingdom) participated in the survey, which included 49 participants from Eastern Europe. This equates to a response rate of 35.0%. Of the respondents, 65.6% classified themselves as ‘senior doctors’ (consultant or above), 24.2% as ‘junior/middle grade doctors’, and 10.2% as ‘other profession’ (mainly comprising microbiologists, molecular biologists, public health professionals, and researchers). Of the respondents, 32.7% stated that they were exclusively managing children with TB, 34.5% exclusively adults with TB, and 32.7% both children and adults. The majority of participants stated that they were working in a university hospital or a regional hospital (58.3% and 20.0%, respectively); few were working in a private practice or primary care setting (0.6% and 4.4%, respectively). A small proportion of participants stated that they were primarily working in a laboratory setting or a public health institution (6.3% and 3.1%, respectively). Participants stated the following specialties to be their main area of work: general paediatrics (3.2%), paediatric pulmonology (12.3%), paediatric infectious diseases (17.6%), general adult/internal medicine (1.6%), adult pulmonology (25.7%), adult infectious diseases (14.4%), microbiology (17.1%), general practice (0.5%); 7.5% of respondents chose ‘other’ (mainly comprising public health, TB laboratories, and research). The majority of participants stated that TB accounted for a large proportion of their average workload [10–25% of their workload (22.2%); 25–50% (12.5%); 50–75% (13.6%); 75–100% (26.7%)]. The majority of respondents stated that they had 20 or more patients with active TB under their care per year on average [5–20 patients (28.7%); 20–50 (21.6%); >50 (29.3%)].

**Figure 1:**
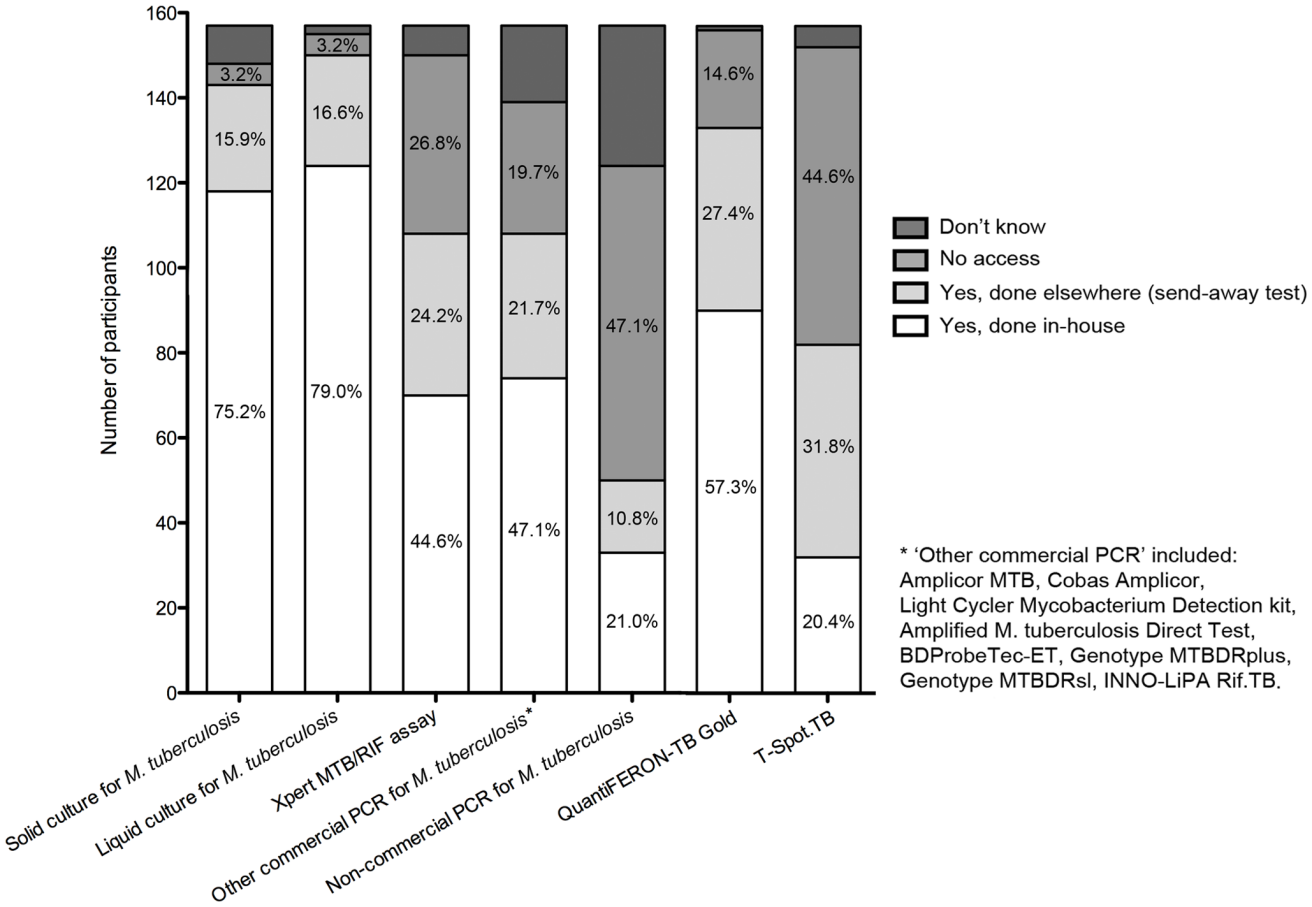
Summary of participants' responses regarding their access to immunological, conventional microbiological and molecular tests for tuberculosis.

Figure 1 summarises the participants' access to a range of microbiological and immunological tests for TB. The vast majority of respondents stated that they had access to both solid and liquid mycobacterial cultures (91.1% and 95.5%, respectively). More than a quarter (26.8%) stated that they did not have access to the Xpert MTB/RIF assay; fewer than half (44.6%) stated that they had access to this assay with the assay being performed at their own institution. Among respondents exclusively providing care for adults, 62.3% stated that they had access to the Xpert MTB/RIF assay (performed at their own institution or as send-away test), while 32.1% stated that they had no access. Among respondents exclusively providing care for children and adolescents, 70.4% stated that they had access to the Xpert MTB/RIF assay, while 24.1% stated that they had no access. There was no statistically significant difference between these two subgroups regarding access to the Xpert MTB/RIF assay (p = 0.39). Among the subgroup of respondents based at a University hospital, 66.7% stated that they had access to the Xpert MTB/RIF assay, while 32.4% stated that they had no access. Among the subgroup of respondents based at a regional hospital or in primary care, 73.7% stated that they had access to the Xpert MTB/RIF assay, while 23.7% stated that they had no access. There was no statistically significant difference between these two subgroups regarding access to the Xpert MTB/RIF assay (p = 0.40). Among the University-based subgroup with access to the Xpert MTB/RIF assay, 62.9% stated the assay was performed at their own institution, while 37.1% stated the assay was performed elsewhere (send-away test); among the regional hospital- and primary care-based subgroup with access to the Xpert MTB/RIF assay, 60.7% stated the assay was performed at their own institution, while 39.3% stated the assay was performed elsewhere (p = 1.00).

Overall, a substantial proportion of respondents stated that they had access to other commercial and/or non-commercial PCR-based assays for TB (68.8% and 31.8%, respectively; see Figure 1 for details regarding ‘other commercial assays’). Only 6.4% of respondents stated that they did not have access to any PCR-based assays for TB; the majority of those respondents were based in Eastern European countries (Bulgaria, Romania, and Ukraine). Fewer respondents stated that they had access (either performed at the same institution or as send-away test) to the T-SPOT.*TB* compared with the QuantiFERON-TB Gold assay (52.2% versus 84.7%).

**Figure 2:**
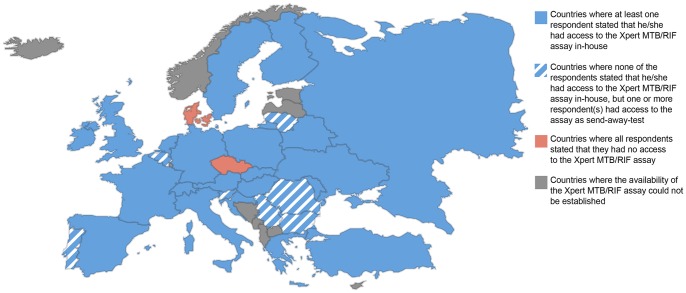
Summary of participants' responses regarding their access to the Xpert MTB/RIF assay according to country.

Figure 2 summarises the participants' responses regarding their ability to access the Xpert MTB/RIF assay according to country. In most countries one or more of the respondents had access to the assay in-house. All respondents from Belgium (n = 5), Lithuania (n = 1), Portugal (n = 2), Romania (n = 7), Serbia (n = 1), and Slovenia (n = 2) stated that they did not have access to the assay in-house; however one or more respondent(s) from those countries stated that they had access to the assay as send-away test, showing that the assay is available at least on a national level. All respondents from the Czech Republic (n = 3) and all respondents from Denmark (n = 5) stated that did not have access to the Xpert MTB/RIF assay, either in-house or as send-away test; however, all Czech and four of the five Danish respondents indicated that they had access to another commercial PCR-based test for TB.

Among participants who had access to the Xpert MTB/RIF assay (n = 109), the majority (95.2%) stated that they had used this assay for the analysis of respiratory samples, 36.5% for pleural fluid, 34.7% for gastric aspirates, 34.7% for cerebrospinal fluid (CSF) samples, 4.3% for stool samples, and 2.6% for blood or serum. In addition, 16.5% stated that they had used the assay for the analysis of ‘other samples’, which included biopsy/tissue samples, lymph node aspirates, joint aspirates and urine samples. The participants who stated that they had used the assay for the analysis of ‘other samples’ (n = 18) were based in a variety of European countries (Belarus, Belgium, Croatia, Finland, France, Italy, the Russian Federation, Slovenia, Spain, Switzerland, Turkey, United Kingdom), with the majority being based at regional or university hospitals (n = 5 and n = 12, respectively).

When asked whether they had ever started a patient on treatment for drug-resistant TB based on history/clinical features and then changed back to treatment for drug-susceptible TB based on an Xpert MTB/RIF assay result suggesting that the organism was susceptible (ie rather than awaiting the results of phenotypic testing), the majority (68.2%) of respondents with access to these assays answered ‘no, never’; a further 19.6% answered ‘yes, but only rarely’. There was no statistically significant difference between Eastern European participants and participants from other parts of Europe (65.6% vs. 69.3% chose ‘no, never’, respectively; p = 0.82). There was also no statistically significant difference between participants based at a University hospital and those based at a regional hospital or in primary care (68.3% vs. 65.4% chose ‘no, never’, respectively; p = 0.81). When asked whether they had ever started a patient on treatment for drug-resistant TB based on an Xpert MTB/RIF assay result suggesting that the organism was resistant (ie rather than awaiting the results of phenotypic testing), 36.9% of respondents with access to these assays answered ‘no, never’, 25.2% ‘yes, but only rarely’, and 30.1% ‘yes, regularly’. There was no statistically significant difference between Eastern European participants and participants from other parts of Europe (36.3% vs. 27.1% chose ‘yes, regularly’, respectively; p = 0.10). There was also no statistically significant difference between participants based at a University hospital and those based at a regional hospital or in primary care (26.2% vs. 42.3% chose ‘yes, regularly’, respectively; p = 0.20).

With regards to funding of the Xpert MTB/RIF or an alternative molecular TB test, respondents most commonly stated that the costs were covered by national insurance/national healthcare cover (37.7%), followed by the hospital (22.6%) and the patient's health insurance (21.8%); a smaller proportion (10.9%) indicated that the costs were covered by a public health service (eg TB control program); none of the participants indicated that the patient himself/herself had to cover the costs.

## Discussion

To our knowledge this is the first study to assess the current landscape of immunological and molecular microbiological assays for the diagnosis of TB across Europe. A number of interesting conclusions can be drawn from the results of this survey. Firstly, in the European setting access to conventional microbiological culture methods and IGRA is widespread. Interestingly, a far greater proportion of survey participants had access to the QuantiFERON-TB Gold assay compared with the T-SPOT.*TB* assay, which may reflect the greater difficulties of integrating an ELISPOT assay into the routine diagnostic laboratory setting.

The majority of participants had access to molecular TB tests. Interestingly, our results show that University hospital-based physicians were no more likely to have access to the Xpert MTB/RIF assay than their colleagues based in the primary care or regional hospital setting. Although a quarter of the participants did not have access to the Xpert MTB/RIF assay, the results show that a large proportion of participants had access to a range of other commercial and non-commercial PCR-based tests. Furthermore, the results show that the Xpert MTB/RIF assay was available to healthcare professionals providing care for patients with TB in most European countries, albeit in some only at regional or supra-regional level (ie as send-away test). This aspect is important, as emerging data suggest that centralised placement of the analytical instrument (eg in a reference laboratory at regional level) may be considerably less useful for patient management than use of the assay in a near-patient setting (ie point-of-care test in a TB outpatient clinic), as the former can extend the time required to produce a test result significantly (potentially until after management decisions have already been made), and poses additional challenges regarding linkage of results [11], [22].

Interestingly, a large proportion of participants had used the Xpert MTB/RIF assay for the analysis of a variety of non-respiratory samples, despite the fact that the assay has been optimised and is solely licensed for the analysis of sputum samples [23]. Importantly, while there are data suggesting that the sensitivity of the Xpert MTB/RIF assay is relatively high with CSF and biopsy samples, previous reports have shown the performance to be suboptimal with stool samples, and pleural, peritoneal and joint fluids [24]-[32]. Notably, the recent WHO policy update on the use of the Xpert MTB/RIF assay for the diagnosis of pulmonary and extrapulmonary TB recommends that the assay should be used in preference to conventional microscopy and culture as initial diagnostic test for CSF specimens in patients presumed to have TB meningitis [33]. In addition, the policy update also recommends the use of this assay as a replacement test for usual practice (including microscopy, culture, and/or histopathology) for testing of lymph node and other tissues in patients presumed to have extrapulmonary TB. However, the document also states that both recommendations are based on very low quality evidence, and highlights that these recommendations do not apply to other biological samples (including stool, urine and blood).

Surprisingly, a large proportion of participants who had access to the Xpert MTB/RIF assay indicated that they were not using the resistance result of the test for clinical management decisions on a regular basis. However, it is difficult to draw firm conclusions from this, as the utility of the Xpert MTB/RIF resistance result depends on the prevalence of rifampicin resistance in the population the test is being applied to (ie greater positive predictive value in populations with high proportions of multidrug-resistant (MDR)-TB). However, our data indicate that participants from Eastern European countries, where the rates of MDR-TB are generally higher than in other European countries [34], [35], are no more likely to base management decisions regularly on the PCR-based resistance result than their colleagues from other parts of Europe. This highlights that further studies are needed to determine how current molecular TB tests impact on patient care and outcome in a routine clinical setting in Europe, particularly in view of a recent study from South Africa that has highlighted the limited impact of the Xpert MTB/RIF assay on management decisions, and ultimately patient outcomes [36].

### Limitations

The response rate in this survey was suboptimal. However, a response rate of 35.0% is considered to be average to good in the context of online surveys [37]. The survey was conducted among healthcare professionals and researchers who are part of a dedicated TB network and have a particular interest and/or expertise in the area of TB, and the data may therefore not be fully representative of the situation in a respective country. Notably, the majority of respondents were working in a tertiary care setting, where the availability of diagnostic tests for TB is likely to be greater than at the primary or secondary care level. Therefore, the results may overestimate the availability of the tests in Eastern European countries, where many TB patients are receiving care outside the tertiary care setting. However, in countries with low TB prevalence the majority of suspected and confirmed cases of active TB are receiving care at tertiary level, supporting the validity of the survey findings particularly in relation to Western and Northern European countries. It is possible that healthcare professionals with particularly high workloads were less likely to participate in this survey, which may have introduced a bias. For some countries the number of survey participants was comparatively small, and some of the data may therefore not be representative of the country as a whole. Nevertheless, the data show that while molecular assays may not be available to some individual healthcare professionals, they are available at least at regional or supra-regional level in most European countries.

### Conclusions

Both immunological and molecular microbiological tests for the diagnosis of TB are widely available across Europe. The QuantiFERON-TB Gold assay is more widely used than the T-SPOT.*TB* assay. The Xpert MTB/RIF assay is available to healthcare professionals in most European countries; the majority of healthcare professionals providing care for TB patients who do not have access to this assay have access to other molecular tests for TB. In clinical practice the Xpert MTB/RIF assay is commonly used for the analysis of non-respiratory samples, despite the fact that the assay is optimised for the analysis of sputum samples. A large proportion of healthcare professionals indicated that they were not using the resistance result of the Xpert MTB/RIF assay for clinical management decisions on a regular basis. Therefore, further research is needed to establish how current molecular TB tests impact on patient care and outcome in a routine clinical setting.

## Supporting Information

Table S1 Summary of the final survey instrument.
(DOCX)

## References

[1] Boehme CC, Nicol MP, Nabeta P, Michael JS, Gotuzzo E, et al (2011) Feasibility, diagnostic accuracy, and effectiveness of decentralised use of the Xpert MTB/RIF test for diagnosis of tuberculosis and multidrug resistance: a multicentre implementation study. Lancet 377: 1495–1505. 21507477 10.1016/S0140-6736(11)60438-8 PMC3085933

[2] Heifets L, Linder T, Sanchez T, Spencer D, Brennan J (2000) Two liquid medium systems, mycobacteria growth indicator tube and MB redox tube, for Mycobacterium tuberculosis isolation from sputum specimens. J Clin Microbiol 38: 1227–1230. 10699027 10.1128/jcm.38.3.1227-1230.2000 PMC86383

[3] Barnard M, Warren R, Gey Van Pittius N, van Helden P, Bosman M, et al (2012) Genotype MTBDRsl line probe assay shortens time to diagnosis of extensively drug-resistant tuberculosis in a high-throughput diagnostic laboratory. Am J Respir Crit Care Med 186: 1298–1305. 23087027 10.1164/rccm.201205-0960OC

[4] Boehme CC, Nabeta P, Hillemann D, Nicol MP, Shenai S, et al (2010) Rapid molecular detection of tuberculosis and rifampin resistance. N Engl J Med 363: 1005–1015. 20825313 10.1056/NEJMoa0907847 PMC2947799

[5] D'Amato RF, Wallman AA, Hochstein LH, Colaninno PM, Scardamaglia M, et al (1995) Rapid diagnosis of pulmonary tuberculosis by using Roche AMPLICOR Mycobacterium tuberculosis PCR test. J Clin Microbiol 33: 1832–1834. 7665654 10.1128/jcm.33.7.1832-1834.1995 PMC228279

[6] Lacoma A, Garcia-Sierra N, Prat C, Ruiz-Manzano J, Haba L, et al (2008) GenoType MTBDRplus assay for molecular detection of rifampin and isoniazid resistance in Mycobacterium tuberculosis strains and clinical samples. J Clin Microbiol 46: 3660–3667. 18784319 10.1128/JCM.00618-08 PMC2576567

[7] Pfyffer GE, Funke-Kissling P, Rundler E, Weber R (1999) Performance characteristics of the BDProbeTec system for direct detection of Mycobacterium tuberculosis complex in respiratory specimens. J Clin Microbiol 37: 137–140. 9854078 10.1128/jcm.37.1.137-140.1999 PMC84189

[8] Viveiros M, Leandro C, Rodrigues L, Almeida J, Bettencourt R, et al (2005) Direct application of the INNO-LiPA Rif.TB line-probe assay for rapid identification of Mycobacterium tuberculosis complex strains and detection of rifampin resistance in 360 smear-positive respiratory specimens from an area of high incidence of multidrug-resistant tuberculosis. J Clin Microbiol 43: 4880–4884. 16145166 10.1128/JCM.43.9.4880-4884.2005 PMC1234138

[9] Vuorinen P, Miettinen A, Vuento R, Hallstrom O (1995) Direct detection of Mycobacterium tuberculosis complex in respiratory specimens by Gen-Probe Amplified Mycobacterium Tuberculosis Direct Test and Roche Amplicor Mycobacterium Tuberculosis Test. J Clin Microbiol 33: 1856–1859. 7665659 10.1128/jcm.33.7.1856-1859.1995 PMC228285

[10] World Health Organization (2010) News release: WHO endorses new rapid tuberculosis test. Available: http://www.who.int/mediacentre/news/releases/2010/tb_test_20101208/en/index.html. Accessed 2014 April 14.

[11] Lawn SD, Mwaba P, Bates M, Piatek A, Alexander H, et al (2013) Advances in tuberculosis diagnostics: the Xpert MTB/RIF assay and future prospects for a point-of-care test. Lancet Infect Dis 13: 349–361. 23531388 10.1016/S1473-3099(13)70008-2 PMC4844338

[12] Lawn SD, Nicol MP (2011) Xpert(R) MTB/RIF assay: development, evaluation and implementation of a new rapid molecular diagnostic for tuberculosis and rifampicin resistance. Future Microbiol 6: 1067–1082. 21958145 10.2217/fmb.11.84 PMC3252681

[13] World Health Organization (2014) WHO monitoring of Xpert MTB/RIF roll-out. Available: http://who.int/tb/laboratory/GeneXpert_rollout_large.jpg. Accessed 2014 April 14.

[14] U.S. Food and Drug Administration (2001) Device approvals and clearances: QuantiFERON -TB - P010033. Available: http://www.fda.gov/MedicalDevices/ProductsandMedicalProcedures/DeviceApprovalsandClearances/Recently-ApprovedDevices/ucm084025.htm. Accessed 2014 April 14.

[15] Tebruegge M, Connell T, Curtis N (2012) Tuberculosis in children. N Engl J Med 367: 1568. 10.1056/NEJMc1210173 23075192

[16] Andersen P, Munk ME, Pollock JM, Doherty TM (2000) Specific immune-based diagnosis of tuberculosis. Lancet 356: 1099–1104. 11009160 10.1016/s0140-6736(00)02742-2

[17] Herrera V, Perry S, Parsonnet J, Banaei N (2011) Clinical application and limitations of interferon-gamma release assays for the diagnosis of latent tuberculosis infection. Clin Infect Dis 52: 1031–1037. 21460320 10.1093/cid/cir068

[18] Tebruegge M, Connell T, Ritz N, Bryant PA, Curtis N (2010) Discordance between TSTs and IFN-gamma release assays: the role of NTM and the relevance of mycobacterial sensitins. Eur Respir J 36: 214–215. 20595171 10.1183/09031936.00025510

[19] Pai M, Dheda K, Cunningham J, Scano F, O'Brien R (2007) T-cell assays for the diagnosis of latent tuberculosis infection: moving the research agenda forward. Lancet Infect Dis 7: 428–438. 17521596 10.1016/S1473-3099(07)70086-5

[20] Dheda K, van Zyl Smit R, Badri M, Pai M (2009) T-cell interferon-gamma release assays for the rapid immunodiagnosis of tuberculosis: clinical utility in high-burden vs. low-burden settings. Curr Opinion Pulm Med 15: 188–200. 10.1097/MCP.0b013e32832a0adc 19387262

[21] Tebruegge M, Salo E, Ritz N, Kampmann B (2013) on behalf of the Paediatric Tuberculosis Network European Trialsgroup (2013) Inclusion of latent tuberculosis infection as a separate entity into the international classification of diseases. Thorax 68: 588. 10.1136/thoraxjnl-2012-202824 23128034

[22] Lawn SD, Kerkhoff AD, Wood R (2012) Location of Xpert(R) MTB/RIF in centralised laboratories in South Africa undermines potential impact. Int J Tuberc Lung Dis 16: 701. 22507934 10.5588/ijtld.12.0131

[23] U.S. Food and Drug Administration (2013) Xpert MTB/RIF assay - decision summary. Available: http://www.accessdata.fda.gov/cdrh_docs/reviews/K131706.pdf. Accessed 2014 April 14.

[24] Tortoli E, Russo C, Piersimoni C, Mazzola E, Dal Monte P, et al (2012) Clinical validation of Xpert MTB/RIF for the diagnosis of extrapulmonary tuberculosis. Eur Respir J 40: 442–447. 22241741 10.1183/09031936.00176311

[25] Hillemann D, Rusch-Gerdes S, Boehme C, Richter E (2011) Rapid molecular detection of extrapulmonary tuberculosis by the automated GeneXpert MTB/RIF system. J Clin Microbiol 49: 1202–1205. 21270230 10.1128/JCM.02268-10 PMC3122824

[26] Moure R, Martin R, Alcaide F (2012) Effectiveness of an integrated real-time PCR method for detection of the Mycobacterium tuberculosis complex in smear-negative extrapulmonary samples in an area of low tuberculosis prevalence. J Clin Microbiol 50: 513–515. 22162564 10.1128/JCM.06467-11 PMC3264142

[27] Nhu NT, Heemskerk D, Thu do DA, Chau TT, Mai NT, et al (2014) Evaluation of GeneXpert MTB/RIF for diagnosis of tuberculous meningitis. J Clin Microbiol 52: 226–233. 24197880 10.1128/JCM.01834-13 PMC3911435

[28] Patel VB, Theron G, Lenders L, Matinyena B, Connolly C, et al (2013) Diagnostic accuracy of quantitative PCR (Xpert MTB/RIF) for tuberculous meningitis in a high burden setting: a prospective study. PLoS Med 10: e1001536. 24167451 10.1371/journal.pmed.1001536 PMC3805498

[29] Porcel JM, Palma R, Valdes L, Bielsa S, San-Jose E, et al (2013) Xpert(R) MTB/RIF in pleural fluid for the diagnosis of tuberculosis. Int J Tuberc Lung Dis 17: 1217–1219. 23827859 10.5588/ijtld.13.0178

[30] Ablanedo-Terrazas Y, Alvarado-de la Barrera C, Hernandez-Juan R, Ruiz-Cruz M, Reyes-Teran G (2013) Xpert MTB/RIF for diagnosis of tuberculous cervical lymphadenitis in HIV-infected patients. Laryngoscope (in press). doi:10.1002/lary.24478 10.1002/lary.24478 24166585

[31] Ligthelm LJ, Nicol MP, Hoek KG, Jacobson R, van Helden PD, et al (2011) Xpert MTB/RIF for rapid diagnosis of tuberculous lymphadenitis from fine-needle-aspiration biopsy specimens. J Clin Microbiol 49: 3967–3970. 21880965 10.1128/JCM.01310-11 PMC3209093

[32] Nicol MP, Spiers K, Workman L, Isaacs W, Munro J, et al (2013) Xpert MTB/RIF testing of stool samples for the diagnosis of pulmonary tuberculosis in children. Clin Infect Dis 57: e18–21. 23580738 10.1093/cid/cit230 PMC3703104

[33] World Health Organization (2013) Xpert MTB/RIF system for the diagnosis of pulmonary and extrapulmonary TB in adults and children – policy update. Available: http://www.stoptb.org/wg/gli/assets/documents/WHO%20Policy%20Statement%20on%20Xpert%20MTB-RIF%202013%20pre%20publication%2022102013.pdf. Accessed 2014 April 14. 25473701

[34] World Health Organization (2013) Global tuberculosis report 2013. Available: http://www.who.int/tb/publications/global_report/en/. Accessed 2014 April 14.

[35] European Centre for Disease Prevention and Control (2013) Tuberculosis surveillance and monitoring in Europe 2013. Available: http://ecdc.europa.eu/en/publications/Publications/Tuberculosis-surveillance-monitoring-2013.pdf. Accessed 2014 April 14.

[36] Theron G, Zijenah L, Chanda D, Clowes P, Rachow A, et al (2014) Feasibility, accuracy, and clinical effect of point-of-care Xpert MTB/RIF testing for tuberculosis in primary-care settings in Africa: a multicentre, randomised, controlled trial. Lancet 383: 424–435. 24176144 10.1016/S0140-6736(13)62073-5

[37] Nulty DD (2008) The adequacy of response rates to online and paper surveys: what can be done? Assess Eval High Educ 33: 301–314.

