# Supplementary material for: Availability and Use of Molecular Microbiological and Immunological Tests for the Diagnosis of Tuberculosis in Europe
Source: PLoS One. 2014 Jun 12;9(6):e99129. doi: 10.1371/journal.pone.0099129 (PMC4055680; doi:10.1371/journal.pone.0099129)
Supplement: Table S1 — Summary of the final survey instrument. (DOCX) [file pone.0099129.s001.docx]

| **Question** | **Answer options** | **Answer text** | **Answer text** |
| --- | --- | --- | --- |
| What is your profession / grade? | Single choice |  |  |
|  |  | Senior doctor (consultant and above) |  |
|  |  | Junior or middle grade doctor |  |
|  |  | Other (please specify) |  |
| What is your main area of work or specialty? | Single choice |  |  |
|  |  | General paediatrics |  |
|  |  | Paediatric pulmonology |  |
|  |  | Paediatric infectious diseases |  |
|  |  | General adult medicine /  Adult internal medicine |  |
|  |  | Adult pulmonology |  |
|  |  | Adult infectious diseases |  |
|  |  | General practitioner |  |
|  |  | Microbiology |  |
|  |  | Other (please specify) |  |
| Which age-group(s) of TB patients do you see/manage? | Single choice |  |  |
|  |  | Children & adolescents (up to 18 years of age) ONLY |  |
|  |  | Adults ONLY |  |
|  |  | All age-groups (ie children, adolescents and adults) |  |
| Where do you mainly work? | Single choice |  |  |
|  |  | Private practice |  |
|  |  | Primary care (public) |  |
|  |  | Regional hospital |  |
|  |  | University hospital |  |
|  |  | Other (please specify) |  |
| In which country do you mainly work? | Single choice |  |  |
|  |  | Dropdown menu of countries |  |
| In your daily practice, how important is tuberculosis in terms of your workload on average? | Single choice |  |  |
|  |  | <10% |  |
|  |  | 10-25% |  |
|  |  | 25-50% |  |
|  |  | 50-75% |  |
|  |  | 75-100% |  |
| On average, how many new patients with active TB per year are under your care? | Single choice |  |  |
|  |  | 1-4 |  |
|  |  | 5-19 |  |
|  |  | 20-50 |  |
|  |  | >50 |  |
| For each test below please indicate whether you have access to the test (ie you can get samples analysed with this test), and whether the test is done at your institution (ie in-house).  (* commercial PCRs include: Amplicor MTB, Cobas Amplicor, Light Cycler Mycobacterium Detection kit, Amplified M. tuberculosis Direct Test, the BDProbeTec-ET, Genotype MTBDRplus, Genotype MTBDRsl, INNO-LiPA Rif.TB,) | Single choice for each item |  |  |
|  |  | QuantiFERON-TB Gold (IGRA) | No access |
|  |  |  | Yes, done in-house |
|  |  |  | Yes, done elsewhere (send-away test) |
|  |  |  | Don’t know |
|  |  | T-Spot.*TB* (IGRA) | No access |
|  |  |  | Yes, done in-house |
|  |  |  | Yes, done elsewhere (send-away test) |
|  |  |  | Don’t know |
|  |  | Solid culture for *M. tuberculosis* | No access |
|  |  |  | Yes, done in-house |
|  |  |  | Yes, done elsewhere (send-away test) |
|  |  |  | Don’t know |
|  |  | Liquid culture for *M. tuberculosis* | No access |
|  |  |  | Yes, done in-house |
|  |  |  | Yes, done elsewhere (send-away test) |
|  |  |  | Don’t know |
|  |  | Xpert MTB/RIF assay | No access |
|  |  |  | Yes, done in-house |
|  |  |  | Yes, done elsewhere (send-away test) |
|  |  |  | Don’t know |
|  |  | Other commercial PCR for *M. tuberculosis ** | No access |
|  |  |  | Yes, done in-house |
|  |  |  | Yes, done elsewhere (send-away test) |
|  |  |  | Don’t know |
|  |  | Other non-commercial PCR for *M. tuberculosis* | No access |
|  |  |  | Yes, done in-house |
|  |  |  | Yes, done elsewhere (send-away test) |
|  |  |  | Don’t know |
| What type of samples have you had analysed by Xpert MTB/RIF?  Please choose all options that apply. | Multiple choices |  |  |
|  |  | Not applicable (I don’t have access to Xpert MTB/RIF testing) |  |
|  |  | Respiratory samples (sputum, nasopharyngeal aspirates, broncho-alveolar lavage fluid) |  |
|  |  | Pleural fluid |  |
|  |  | Gastric aspirate |  |
|  |  | Serum / blood |  |
|  |  | Cerebrospinal fluid |  |
|  |  | Stool |  |
|  |  | Other (please specify) |  |
| Have you ever started a patient on treatment for drug-resistant TB based on history/clinical features and then changed back to treatment for drug-susceptible TB based on an Xpert MTB/RIF result suggesting that the organism is susceptible (ie rather than having waited for the results of phenotypic testing)? | Single choice |  |  |
|  |  | Not applicable (I don’t have access to Xpert MTB/RIF testing) |  |
|  |  | No, never |  |
|  |  | Yes, but only rarely |  |
|  |  | Yes, regularly |  |
|  |  | Yes, always |  |
| Have you ever started a patient on treatment (or changed a patient to treatment) for drug-resistant TB based on an Xpert MTB/RIF result suggesting that the organism is resistant (ie rather than having waited for the results of phenotypic testing)? | Single choice |  |  |
|  |  | Not applicable (I don’t have access to Xpert MTB/RIF testing) |  |
|  |  | No, never |  |
|  |  | Yes, but only rarely |  |
|  |  | Yes, regularly |  |
|  |  | Yes, always |  |
| Who typically carries the costs of the Xpert MTB/RIF test (or an alternative molecular test for TB you have access to)?  Please choose only one option. | Single choice |  |  |
|  |  | Not applicable (I don’t have access to Xpert MTB/RIF or other molecular tests) |  |
|  |  | The patient |  |
|  |  | The hospital |  |
|  |  | The patient’s health insurance (public or private) |  |
|  |  | The national health insurance (e.g. UK NHS or similar national healthcare cover) |  |
|  |  | A public health service (eg TB control programme) |  |
|  |  | Alternative public funding / government |  |
|  |  | Research funding |  |
|  |  | Don’t know |  |
|  |  | Other (please specify) |  |
